# [Retracted] MicroRNA-320a downregulation mediates human liver cancer cell proliferation through the Wnt/β-catenin signaling pathway

**DOI:** 10.3892/ol.2025.15284

**Published:** 2025-09-22

**Authors:** Caicheng Lu, Zengwei Liao, Minxian Cai, Guirong Zhang

Oncol Lett 13: 573–578, 2017; DOI: 10.3892/ol.2016.5479

Following the publication of this paper, it was drawn to the Editor's attention by a concerned reader that, regarding the colony formation assay data shown in Fig. 2E on p. 575. two of the images after a rotation were found to be more similar than expected. Upon performing an independent analysis of this figure in the Editorial Office, it subsequently came to light that three of the four colony formation assay images apparently contained data in common, albeit with different rotations of the images, and moreover, specific groupings of cells appeared to be absent in certain zones of the images, suggesting that these images may have been inappropriately edited.

Considering the results obtained from the analysis of this figure, the Editor of *Oncology Letters* has decided that this paper should be retracted from the Journal. The authors were asked for an explanation to account for these concerns, but the Editorial Office did not receive a reply. The Editor apologizes to the readership for any inconvenience caused.

